# Sensing Antibiotics in Wastewater Using Surface-Enhanced Raman Scattering

**DOI:** 10.1021/acs.est.3c00027

**Published:** 2023-03-19

**Authors:** Yen-Hsiang Huang, Hong Wei, Peter J. Santiago, William John Thrift, Regina Ragan, Sunny Jiang

**Affiliations:** † Department of Civil and Environmental Engineering, University of California, Irvine, Irvine, California 92697, United States; ‡ Department of Materials Science and Engineering, University of California, Irvine, Irvine, California 92697, United States

**Keywords:** quinoline, SERS, Self-Assembled SERS substrate, SERStrate

## Abstract

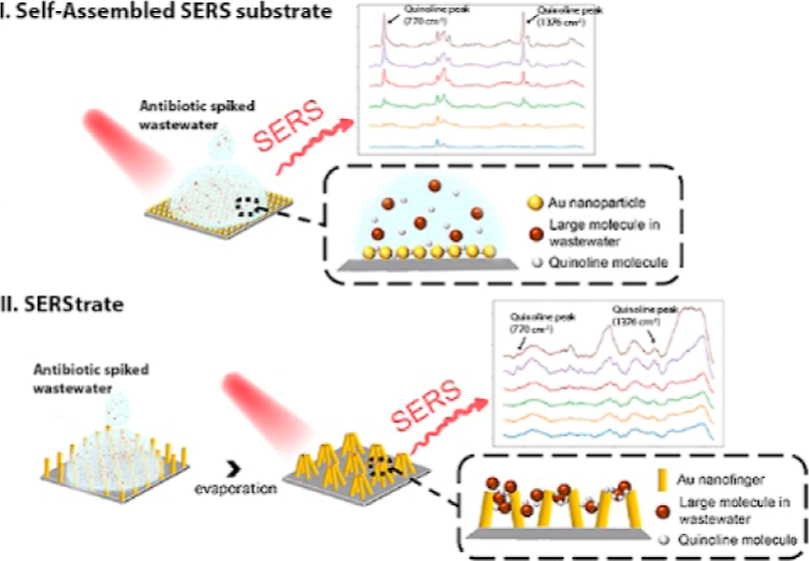

Rapid and cost-effective detection of antibiotics in wastewater and through wastewater treatment processes is an important first step in developing effective strategies for their removal. Surface-enhanced Raman scattering (SERS) has the potential for label-free, real-time sensing of antibiotic contamination in the environment. This study reports the testing of two gold nanostructures as SERS substrates for the label-free detection of quinoline, a small-molecular-weight antibiotic that is commonly found in wastewater. The results showed that the self-assembled SERS substrate was able to quantify quinoline spiked in wastewater with a lower limit of detection (LoD) of 5.01 ppb. The SERStrate (commercially available SERS substrate with gold nanopillars) had a similar sensitivity for quinoline quantification in pure water (LoD of 1.15 ppb) but did not perform well for quinoline quantification in wastewater (LoD of 97.5 ppm) due to interferences from non-target molecules in the wastewater. Models constructed based on machine learning algorithms could improve the separation and identification of quinoline Raman spectra from those of interference molecules to some degree, but the selectivity of SERS intensification was more critical to achieve the identification and quantification of the target analyte. The results of this study are a proof-of-concept for SERS applications in label-free sensing of environmental contaminants. Further research is warranted to transform the concept into a practical technology for environmental monitoring.

## Abstract 2

This study demonstrates the rapid detection of quinoline, a small-molecular-weight antibiotic, in wastewater using surface-enhanced Raman scattering.

## 1 Introduction

Intensive use of antibiotics in clinical and agricultural applications has led to the discharge of large quantities of antibiotics into sewer systems.^1^ Wastewater treatment plants (WWTPs) serve not only as collection points for antibiotics from sewer networks but also play an important role in degrading and removing them before the water is discharged into the environment or is supplied for various reuse applications. However, several studies have indicated that removal efficiencies of antibiotics in WWTPs are usually low.^2−4^ The wastewater antibiotics may also amplify the antibiotic-resistant bacteria (ARB) and antibiotic-resistant genes (ARG) during biological wastewater treatment^5^ such that treated wastewater becoming an important source of antibiotics, ARB, and ARG entering the environment and reused water. Antibiotics in an aquatic environment, even at low concentrations, can promote the breeding of ARB through mutation or horizontal gene transfer, allowing microorganisms to survive in the presence of antibiotics.^6^ Therefore, residual antibiotics in treated wastewater for surface discharge have the potential to adversely affect ecosystems. Residual antibiotics in reused water may have a direct impact on human health by inducing antibiotic-resistant infections.^7^ The World Health Organization (WHO) has listed antibiotic resistance as one of the biggest threats to global health, food security, and development.^8^ Moreover, the US Centers for Disease Control and Prevention (CDC) estimates that at least 2.8 million people get an antibiotic-resistant infection, and more than 35,000 people die from such infections annually in the United States.^9^

Rapid and cost-effective detection of antibiotics in wastewater and through the wastewater treatment processes is an important first step in developing more effective strategies for their removal. Numerous efforts have been devoted to developing robust analytical techniques for antibiotic detection and quantification. Instrument-based methods such as capillary electrophoresis, high-performance liquid chromatography, liquid chromatography–mass spectrometry (LC–MS), and liquid chromatography–tandem mass spectrometry (LC–MS/MS) are the well-established analytical methods.^10,11^ However, the instrumentation cost is high, and the analytical procedures require intensive sample preparation steps by well-trained laboratory personnel.^12^ Particularly, the pre-treatment and pre-concentration processes during sample preparation are time-consuming. There is a significant time delay from sample collection to results. Detection by the enzyme-linked immunosorbent assay (ELISA) relies on the highly specific antigen–antibody interaction to capture and detect target antibiotics in samples. However, ELISA suffers from cross-reaction with interferences in environmental sample matrices, significantly lessening the accuracy and selectivity of the test.^13^ More recently, new analytical methods such as electrochemical, colorimetric, and fluorescence sensors are being developed for antibiotic monitoring in the environment.^14−16^ These methods have reported a relatively short response time, ease of use, portability, and sufficient sensitivity and accuracy,^16^ but their performances in the complex environmental matrix remain to be validated. In recent studies, optical and electrochemical sensing platforms have also been coupled with aptamers, known as aptasensors,^17^ which use either RNA or DNA aptamers for specific binding of target antibiotics. Despite the promise of aptasensors in detecting specific antibiotics in a wide range of matrices with minimum sample preprocessing, aptasensors are challenged by the aptamer design process. In addition, because of nonspecific interactions with interferences in the environmental matrix, the sensitivity of the aptasensors is relatively poor in natural water matrices.^18^ Hence, the selectivity of the aptasensors in environmental matrices still needs to be further evaluated.

Vibrational spectroscopy techniques, specifically surface-enhanced Raman scattering (SERS), have attracted considerable attention for antibiotic detection.^19−24^ SERS is a highly sensitive technique that provides information about the molecular structure via the vibrational spectra of molecular bonds. Raman signals can be enhanced significantly when the molecules are attached to rough metal surfaces or nanostructures because of electromagnetic enhancement and the chemical charge-transfer effect.^25^ SERS has been shown to identify chemical and biomedical species at parts per billion (ppb) levels or even single molecules.^26^ For example, Dhakal et al. reported a label-free SERS method for the screening of tetracycline in whole milk. Although several tetracycline peaks overlap with those of milk, they found that a few tetracycline peaks were unique for tetracycline identification.^27^ Therefore, SERS has the potential to serve as a label-free online sensor to identify specific molecules, including antibiotics.

Despite promises, unlike the relatively simple composition of milk where usually a number of known proteins and lipids are predominant, the complexity of environmental samples may yield unpredictable overlapping spectra that can interfere with the Raman signals of the target chemicals. Moreover, the interference species in wastewater can hinder the target chemical from attaching to hotspots (regions of field enhancements) on metal surfaces or nanostructures.^28,29^ One strategy to overcome the interference is to pre-tag SERS reporters that have a higher selectivity for target molecules. For instance, antibody-based SERS reporters can capture the target biomolecules through antibody–antigen interactions, while aptamer-based SERS reporters focus on the selective enhancement of targets including metal ions, proteins, nucleic acids, viruses, cells, and even bacteria.^30^ However, the analytical methods using SERS reporters are limited to pre-determined target species and require considerable modification of SERS substrates.^31^ Developing SERS reporters for rapid, highly efficient, and specific capture of target molecules that also achieve a high SERS is also no small feat. Thus, label-free SERS is a preferred option for target detection.

Previously, we reported the fabrication of SERS substrates using the chemical assembly of gold nanoparticles from colloids using electrohydrodynamic flow and the creation of two-dimensional arrays of discrete nanoparticle clusters. Our design of driving chemical reactions between ligands on nanoparticles (self-assembly) allows for the precise control of nanogap spacing. This is advantageous for controlling near-field optical properties, exhibiting reproducible billion-fold signal enhancement in Raman measurements.^32^

Here, we report testing and comparing of two surfaces of gold nanostructures as SERS substrates for label-free capture and Raman signal enhancement of quinoline, a small-molecular-weight antibiotic that is commonly found in wastewater. Quinoline is selected as a model molecule because of its molecular size and ring structure, which is favorable for SERS detection. The self-assembled SERS substrate, as previously reported, was fabricated using a hierarchical chemical assembly method to control sub-nano gap spacings.^32^ The second SERS substrate was purchased from Silmeco (Denmark) and is a commercial product with gold nanofingers. The study tests the hypothesis that nanogaps on the self-assembled SERS substrate have specific selectivity based on the molecular size, which excludes the interference from large molecules that are commonly found in wastewater samples. The study showed a rapid detection of quinoline molecules in wastewater on the self-assembled SERS substrate in the concentration spanning 5 orders of magnitude from 50 ppm down to 5 ppb in the presence of diverse organic and inorganic contaminants. The results from this proof-of-concept study indicate the potential for real-time, label-free sensing of antibiotics in wastewater.

## 2 Materials and Methods

### 2.1 Chemicals and Wastewater Samples

Quinoline was used as the target analyte to evaluate the capability of label-free signal quantification using SERS in wastewater. 98% reagent grade quinoline (C_9_H_7_N, 129.16 g/mol) was purchased from Sigma-Aldrich (St. Louis, MO). A 50 ppm quinoline stock solution was prepared by diluting quinoline in nanopore deionized (DI) water (Milli-Q Millipore System).

To test the selectivity of SERS on the substrate, glycine (C_2_H_5_NO_2_, 75.07 g/mol), l-arginine (C_6_H_14_N_4_O_2_, 174.2 g/mol), erythromycin (C_37_H_67_NO_13_, 733.93 g/mol), humic acid (C_187_H_186_O_89_N_9_S_1_, 4015.55 g/mol), and microcystin-LR (C_49_H_74_N_10_O_12_, 995.19 g/mol) were included as the reference molecules. Erythromycin and glycine were purchased from Sigma-Aldrich (St. Louis, MO). Humic acid and microcystin-LR were purchased from Fisher Scientific (Pittsburgh, PA). l-Arginine was purchased from Alfa Aesar (Haverhill, MA). Each reference chemical was dissolved in nanopore DI water to prepare a 5 ppm stock solution.

Wastewater samples collected from a local WWTP were used as the background sample matrix to examine the SERS signal intensification and interferences from organic and inorganic molecules in sewage. The wastewater was treated by advanced primary sedimentation followed by an activated sludge process with nitrification and denitrification. The secondary effluent used in this study meets the wastewater discharge requirement for biochemical oxygen demand (BOD), trace organics, and metal water quality parameters^33^ and is treated further for indirect potable water reuse.^34^ The range of water quality parameters in the secondary effluent is provided in the Supporting Information (Table S1). Although trace antibiotics had been reported in secondary wastewater effluents,^35,36^ the presence of quinoline in the secondary wastewater effluent from the specific plant has not been reported. The annual total organic carbon and total dissolved solids in the secondary effluent from this plant is 14 and 935 mg/L, respectively, suggesting the presence of interference organic and inorganic molecules in the secondary effluent (Table S1).

### 2.2 SERS Substrates

**Figure 1:**
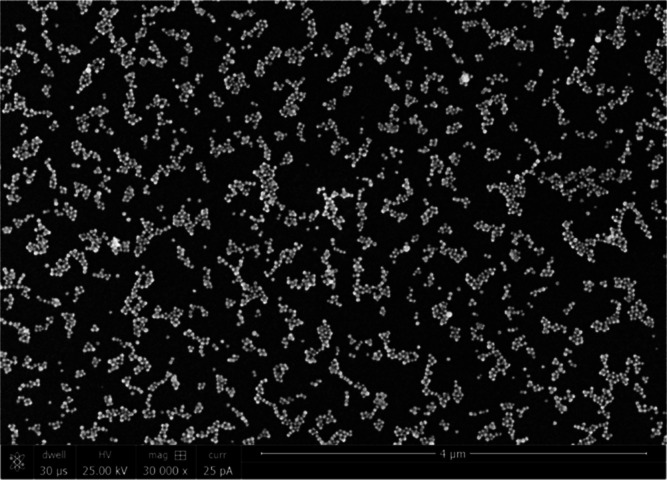
SEM image of the self-assembled SERS substrate.

Self-assembled SERS substrates were fabricated in microfluidic channels with a capacitor architecture. In brief, silicon substrates (NOVA Electronic Materials) were spin coated with poly (styrene-*b*-methyl methacrylate) (PS-*b*-PMMA) thin films as described in a previous work^37^ to serve as the working electrode. Indium tin oxide-coated glass slides (Delta Technologies) served as the counter electrode. Electrical contacts were made by platinum wires and silver paste (Epoxy Technology). 20 μL of 2.6 nM lipoic acid-functionalized Au nanoparticles (NPs, 40 nm, Nanocomposix) along with freshly prepared N-hydroxysulfosuccinimide (s-NHS, Sigma-Aldrich) and 1-ethyl-3-(3-dimethyl aminopropyl) carbodiimide (EDC, Sigma-Aldrich) solution were placed inside a microfluidic channel for chemical cross-linking reactions as described in a previous work.^32^ An oscillation electric field with a potential of 5 Vp and a frequency of 100 Hz was applied to the microfluidic channel for 2 min to deposit a Au-NP seed layer; then the second deposition step was conducted with the same potential but with a frequency of 1000 Hz for 2 min. After each deposition step, the electrode cell was dismantled, and the sensor surface was thoroughly rinsed with DI water and isopropyl alcohol (IPA, Sigma-Aldrich) and then dried with N_2_. Chemical cross-linking reactions between NPs leads to Au-NP clusters with a reproducible SERS signal over a large area.^32^ A scanning electron microscopy (SEM) image of a self-assembled SERS surface is depicted in Figure 1. The observed gap spacing is 0.9 nm with a high uniformity. A normalized SERS intensity map of benzenethiol’s vibrational band acquired over a 100 × 100 μm area was shown in our previous report.^38^ The SERS intensity has a relative standard deviation (RSD) of 10.4%, indicating the uniform SERS enhancements on the self-assembled SERS substrates. Detailed characterizations of the self-assembled SERS substrate including preparation repeatability and reproducibility of signal can be found in the previous reports.^32,38^

SERStrates, the commercially available SERS substrates with gold nanopillars, were purchased from Silmeco (Denmark). According to Silmeco, SERStrate has a nanofinger design that is used to capture molecules and create hotspots via the leaning process happening with solvent evaporation. The structure of SERStrate is available from the company’s website.^39^ SERStrate has been used for the detection of trace amounts of explosives as well as chemical warfare agents, according to the manufacturer’s website.^39^

### 2.3 Sample Preparation

Before each experimental trial, quinoline working solutions in the concentration range from 5 ppm to 5 ppb were freshly prepared by diluting the stock solution with DI water. Wastewater samples were spiked with quinoline in the same concentration range to determine the sensitivity and interference of chemical species in wastewater matrices for SERS.

For Raman spectral analysis on the self-assembled SERS substrate, 10 μL of a solution of the analyte was spotted directly onto the substrate for immediate Raman measurements without the need of drying. After Raman measurements, the sample droplet was blown off from the substrate surface by pressured air; the surface was washed by DI water and reused for the next sample to simulate the near real-time measurement of quinoline in the wastewater stream. Quinoline-spiked samples were tested from a low concentration to a high concentration in the serial diluted samples.

For Silmeco SERStrate, the same volume of the analyte solution was loaded onto the substrate. Since SERStrate relies on solvent evaporation to create leaning of gold fingers to shrink the nanogap space for SERS, the loaded samples were dried at room temperature for 40 min to evaporate water before Raman measurement, following the manufacturer’ protocols.

To test the selective intensification of target molecules on the self-assembled SERS substrate, 10 μL of 5 ppm quinoline, glycine, l-arginine, humic acid, microcystin-LR, and erythromycin solution were loaded onto the self-assembled SERS substrate for Raman measurement following the same procedure as for quinoline.

### 2.4 Reusability of the SERS Substrate

The cleaning procedure for the self-assembled SERS substrate was carried out following the protocol described in a previous study.^38^ In brief, the used substrate was rinsed and soaked in 50 mL of DI water for 20 min, then air dried at the end of DI water soaking and re-examined for Raman spectra to monitor the quinoline signal intensity. Quinoline Raman peaks collected using freshly made and DI water-washed self-assembled SERS substrates were compared to evaluate the reusability of the self-assembled SERS substrate.

DI water washing as performed for the self-assembled SERS substrate could not remove the existing chemical signals from SERStrate because of the trapping of dried chemicals within the nanofingers. A more aggressive procedure to remove organics was used to test the reusability of SERStrate. In brief, the used SERStrate was rinsed by DI water, then washed with 5 N HCl for 10 min to create an acidic environment for oxidizing carbonaceous molecules. Following the acid wash, SERStrate was rinsed with 70% ethanol for 30 s, followed by 10 s of DI water rinse. The DI water was blown off by pressured air and dried. The cleaned SERStrate was examined for residual quinoline signal before being reused for the second round of quinoline testing.

### 2.5 Raman Measurements on the SERS Substrate and UPLC–MS/MS Analysis

Raman spectra were collected using a Renishaw InVia Raman microscope (Renishaw, Wotton-under-Edge, UK) coupled with a 785 nm excitation wavelength laser. For droplet measurement using the self-assembled SERS substrate, a 60× water immersion objective lens with a 1.2 numerical aperture was used for Raman spectra collection. The measurements were taken with 7.3 μW laser power and 0.5 s exposure time, scanning a spectral region from 508 to 1640 cm^–1^.

For Raman measurement using the Silmeco SERStrate, a 50× objective lens was coupled with 7.3 μW laser power and 0.5 s exposure time to collect Raman spectra. Multiple Raman spectral measurements were collected from each sample using the simple mapping measurement method (Renishaw, UK). Pixels with 4 μm step size were generated within a 100 × 100 μm^2^ area on the SERS substrate. A complete spectrum was acquired at each pixel. A total of 625 Raman spectra collected within 5 min were used in modeling and analysis. To validate the SERS assay, a Quattro Premier UPLC–MS/MS instrument coupled with an Acquity BEH C18 UPLC column (Waters Corp, Milford, MA) was applied to quantify the concentration of quinoline.

### 2.6 Spectral Preprocessing

Raman spectra were preprocessed, analyzed, and visualized using Python 3.6.6. The details of the processing steps were presented in the previous studies.^26,40^ In brief, background subtraction was first carried out using the asymmetric least-squares (AsLS) method^41^ in NumPy with λ = 10,000, *p* = 0.001 to extract the true Raman peak intensities. Numerical processing was conducted using the Savitzky–Golay algorithm^42^ with a third-order polynomial and a window size of 11 for Raman spectra, smoothing to increase the precision of the data without distorting the signal tendency. Outlier elimination was performed using the Isolation Forest algorithm in Scikit-Learn to isolate the misleading Raman data caused by background fluorescence, contamination, or poor focusing. Finally, Raman spectra were scaled to have a minimum value of 0 and a maximum value of 1 with MinMaxScaler implemented in Scikit-Learn. The preprocessing allows for the comparison of measurements with slight intensity deviations due to the experimental setup.

### 2.7 Spectral Analysis and Modeling

According to a literature report, the Raman spectra of the quinoline within the region from 700 to 1640 cm^–1^ include several characteristic peaks locating at 760, 1014, 1034, 1372, 1392, 1433, and 1571 cm^–1^.^43^ The peak at 760 cm^–1^ represents ring deformation. The peaks at 1014 and 1034 cm^–1^ are attributed to ring breathing. CCC stretching modes contribute to the peaks at 1372, 1392, and 1571 cm^–1^. CH rocking modes give rise to the peak at 1433 cm^–1^.^44−46^ Two peaks at 760 and 1372 cm^–1^ are the most intense among all peaks. For quinoline quantification, the Raman peak at 770 cm^–1^ (a shift from 760 cm^–1^) was first used for signal quantification. The relationship between this single quinoline peak intensity from the averaged Raman spectra and the sample concentration was evaluated using the linear regression algorithm in Microsoft Excel. *R*^2^ of the linear regression was calculated to present the sensitivity and accuracy of the detection method using the single peak intensity. Limit of detection (LoD) was calculated using the equation LoD = 3*S*_a_/*b*,^47^ where *S*_a_ is the standard deviation of the Raman peak intensities at 770 cm^–1^ in the measurements for the blank sample and *b* is the slope of the linear regression curves.

To further increase the detection sensitivity and minimize the background interference to the target Raman spectra, a predictive model was developed using the non-negative matrix factorization (NMF) method^48^ followed by partial least squares (PLS) regression analysis,^49^ as previous described.^38,40^ In brief, the model first applied the NMF algorithm to differentiate the vibrational spectra of quinoline from interference species in wastewater and the SERS substrate. NMF was implemented with Scikit-Learn using default settings to extract quinoline characteristic components that were decomposed from the complete Raman spectra. PLS regression, which combines the characteristics of principal component analysis (PCA) with multiple linear regression to predict a set of dependent variables from a large set of independent variables, was also applied with Scikit-Learn. Consequently, PLS analyzed the full quinoline component extracted with NMF to build a predictive model of quinoline concentration in a complex matrix. The model was constructed using 80% of the spectral data, and the remaining 20% of the spectra were used to evaluate the accuracy of the model. PCA^50^ was performed on the SERS data collected from different samples using Scikit-Learn to visualize the difference of each sample by decreasing the dimensional variables. For each sample, 50 Raman spectra were randomly selected and displayed on a coordinate system.

## 3 Results

### 3.1 Detection of Quinoline in Pure Water

**Figure 2:**
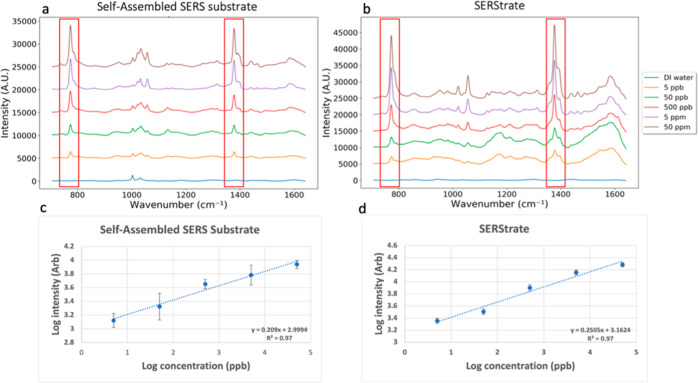
SERS spectra of DI water and quinoline-spiked DI water in the concentration range between 5 ppb and 50 ppm collected using the (a) self-assembled SERS substrate and (b) SERStrate. Relationship between the log values of Raman intensity of the quinoline peak at 770 cm^–1^ and log values of quinoline concentration using the (c) self-assembled SERS substrate and (d) SERStrate.

Averaged Raman spectra of quinoline in DI water collected using the self-assembled SERS substrate at concentrations of 0, 5 ppb, 50 ppb, 500 ppb, 5 ppm, and 50 ppm are shown in Figure 2a. The experimental spectra locating at 770, 1019, 1030, 1376, 1391, 1440, and 1579 cm^–1^ were in good agreement with the quinoline characteristic peaks shown in the literature report,^43^ while the peaks locating at 1057, 1133, 1264, 1314 and 1463 cm^–1^ were not previously reported in the literature. The peaks at 1057, 1133, and 1314 cm^–1^ are attributed to CH bending. The peak at 1264 cm^–1^ represents CNC bending, and the peak at 1463 can be assigned to CH rocking.^44−46^ The quinoline peak assignments are summarized in the Supporting Information (Table S2). Two non-quinoline peaks at 1002 and 1145 cm^–1^ found in the experimental spectra could be contributed to the residual of methanol used to clean the microscope objective lens as their peak intensity did not increase with the increase of quinoline concentration. Quinoline Raman peaks located at 770 and 1376 cm^–1^ are the two most intense peaks, and the intensity increased with the increase of quinoline concentration from 5 ppb to 50 ppm. Figure 2c demonstrates the linear relationship between the log10 transformed quinoline concentration and the log10 Raman intensity at 770 cm^–1^. The linear regression equation log *C* = 0.209 log *I* + 2.9994 was established using the data from the quinoline samples of 5 ppb to 50 ppm with the *R*^2^ of 0.97, where *C* and *I* represent quinoline concentration and Raman spectral intensity at 770 cm^–1^, respectively. The results of quinoline detection using UPLC–MS/MS show a similar detection range of quinoline concentrations, indicating the validity of SERS quantification (Figure S1).

Similarly, quinoline in DI water was also captured by SERStrate (Figure 2b). Similar to the spectra collected using the self-assembled substrate, characteristic quinoline peaks at 770 and 1376 cm^–1^ were clearly observed on SERStrate down to the concentration of 5 ppb (Figure 2b). The linear regression equation log *C* = 0.2505 log *I* + 3.1624 calculated using log10 transformed quinoline concentration and Raman spectral intensity at 770 cm^–1^ showed a strong correlation with *R*^2^ = 0.97 (Figure 2d). SERStrate and the self-assembled SERS substrate had a similar sensitivity to effectively detect and quantify quinoline in pure water with the LoD of 1.15 and 2.57 ppb, respectively. The RSD of peak intensities was larger in the measurements by the self-assembled SERS substrate, which is attributed to the molecular diffusion around the hotspots when collecting signals in the wet mode.

### 3.2 Detection of Quinoline in Wastewater

**Figure 3:**
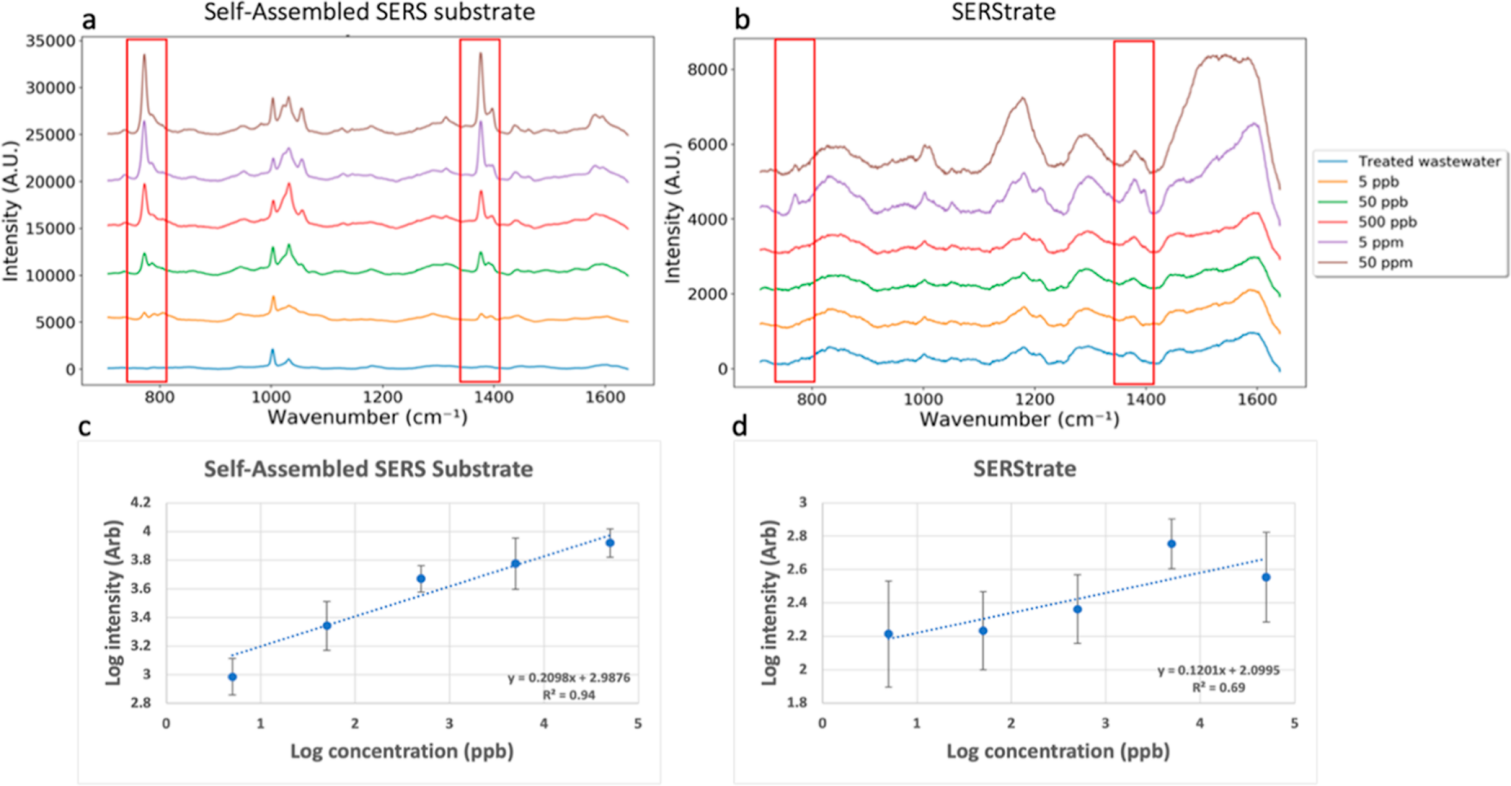
SERS spectra of treated wastewater and quinoline-spiked wastewater in the concentration range between 5 ppb and 50 ppm collected using the (a) self-assembled SERS substrate and (b) SERStrate. Relationship between the log values of Raman intensity of the quinoline peak at 770 cm^–1^ and log values of quinoline concentration using the (c) self-assembled SERS substrate and (d) SERStrate.

When the vibrational spectra of quinoline-spiked wastewater was characterized on the two different SERS substrates, two very different outcomes were observed (Figure 3). Similar Raman spectra as seen for pure water samples were observed on the self-assembled substrate (Figure 3a). Characteristic quinoline peaks at 770 and 1376 cm^–1^ (shift from 760 and 1372 cm^–1^) were clearly seen at seeding concentrations between 5 ppb and 50 ppm. When comparing the spectra of 5 ppb quinoline-spiked DI water, the quinoline peak intensity of 5 ppb quinoline-spiked wastewater was slightly lower. This might be a consequence of the adsorption of a small amount of quinoline to large organic molecules and debris in wastewater, which prevents quinoline access to the SERS hotspot. In addition, Raman spectra of quinoline-spiked wastewater exhibited greater RSDs compared with the spectra of quinoline-spiked DI water, suggesting some interference of sewage molecules in the Raman spectra.

Examination of the wastewater only control sample revealed similar Raman spectra as those for pure water on the self-assembled substrate; no other visible peak was found in the Raman spectra other than the substrate background peaks. This result suggests that the wastewater organics and inorganics had a minimal impact on the Raman spectra in the region because sewage molecules were not intensified by the self-assembled SERS substrate (Figure 3a). The relationship between log10 quinoline concentration and log10 Raman intensity at 770 cm^–1^ collected from wastewater samples is plotted in Figure 3c. A linear relationship with the *R*^2^ of 0.94 was identified. The linear regression curves were nearly identical for quinoline in wastewater and in DI water, while the actual peak intensity of 5 ppb quinoline in treated wastewater was slightly lower than the intensity estimated by the linear regression curves. Furthermore, the SERS measurement of this scenario yields a LoD of 5.01 ppb, which is slightly higher than the LoD of the pure water scenario. The results suggest that the complex matrix did not significantly interfere with the Raman spectra of quinoline on the self-assembled SERS substrate at the high concentration range, but it had a slight effect on the quantification accuracy at the concentration of 5 ppb when using a single characteristic Raman peak to detect quinoline concentrations.

Raman spectra of quinoline-spiked wastewater collected on SERStrate (Figure 3b) only showed discernible quinoline peaks at 770 and 1376 cm^–1^ in the two samples with the highest spiked concentrations of 5 and 50 ppm. The characteristic Raman peaks were not observed at seeding concentrations below 500 ppb (Figure 3b). There were high background spectral noises detected for the control wastewater. The noise spectra were enhanced when the quinoline-spiked wastewater samples were loaded on SERStrate from the low to the high concentration, suggesting that Raman signals of wastewater molecules were captured and intensified during the drying process. In comparison with results for quinoline spiked in pure water, the signal intensity of quinoline peaks was significantly reduced (Figure 3d) due to the interference from wastewater molecules that likely blocked the SERS hotspots. There was no linear relationship between the log10 Raman intensity of the quinoline peak at 770 cm^–1^ and the log10 quinoline concentration (Figure 3d). In fact, the Raman signal intensity for quinoline concentration of 50 ppm was lower than its intensity at 5 ppm (Figure 3d), suggesting interference of sewage molecules likely by occupying the nanogap space and blocking the access of quinoline to the hotspots. Moreover, the Raman spectral intensities collected from the randomly selected spots on the 100 × 100 μm^2^ SERStrate were highly heterogeneous as shown by the large RSDs in the measurements for each sample (Figure 3d). The heterogeneity could be the result of the “coffee ring effect”^51^ created during drying of samples on SERStrate. The poor sensitivity and high heterogeneity in this detection scenario led to a LoD of 97.5 ppm, around 10,000-fold higher compared to the LoD of the pure water setting (1.15 ppb) and even higher than the maximum spiked concentration.

When applying PCA to Raman spectral data in an attempt to further classify the spectra from the target and interferences, the results showed that Raman spectra of treated wastewater were very similar to those of pure water on the self-assembled substrate (Figure S2a). The spectra observed from the quinoline negative control samples reflected the spectra of water and the self-assembled substrate. Specifically, the Raman spectra of the contaminants in treated wastewater were not observed due to the lack of Raman signal amplification of large molecules in the small nanogaps. A few outliers of the wastewater control measurements were observed in the PCA plot, which may be due to variability of the substrate surface or detection of molecules in the background of the wastewater. The Raman spectra of the samples spiked with 50 ppm quinoline in either pure water or wastewater overlapped on the PCA plot, indicating similar Raman spectral profiles of the two samples on the self-assembled SERS substrate.

On the other hand, the PCA plot of the Raman spectra of quinoline in DI water and in wastewater on SERStrate were very different (Figure S2b). First, a separation between Raman spectra of wastewater and pure water was found, suggesting that molecules in wastewater were intensified by SERStrate. The quinoline spectra of the samples spiked with 50 ppm quinoline in DI water and wastewater formed separate clusters on the PCA plot, indicating the drastically decreased intensity of quinoline signals and the high background signal from the molecules in wastewater.

SERStrate and the self-assembled substrate are designed differently for achieving SERS. SERStrate requires drying to trap molecules within the nanofingers. Therefore, there is no selectivity for the molecular size of the target. Wastewater molecules, regardless of size, can be trapped by the nanofingers and intensified. The self-assembled substrate enhances spectral signals from molecules within the designed nanogap space; therefore, small molecules on length scales of the nanogap distance can enter the gap space and be disproportionally enhanced. Large molecules in wastewater, for example, humic acid (General molecular weight: 4015.55 g/mol), did not interfere with the signal from the Raman spectra of the small-molecular-weight quinoline (Figure S2). In fact, insignificant Raman signals were observed for larger molecular-weight chemicals such as erythromycin (733.93 g/mol), microcystin-LR (995.189 g/mol), and humic acid (4015.55 g/mol), according to our screening tests (Figure S3), suggesting that these large molecules may be excluded from approaching the nanogap space between gold nanoparticles. In addition to the large molecules, testing of the interferences from several small molecules that are commonly found in wastewater such as glycine and l-arginine also showed negligible results (Figure S3). This is likely because their molecular structures have a very small Raman cross section. Although these small molecules can enter the nanogap space between the gold nanoparticles, they do not interfere with the signal from the Raman spectra of quinoline, especially at the concentration of 5 ppm or less.

### 3.3 Quantitative Detection of Quinoline in Wastewater Using a Predictive Model

**Figure 4:**
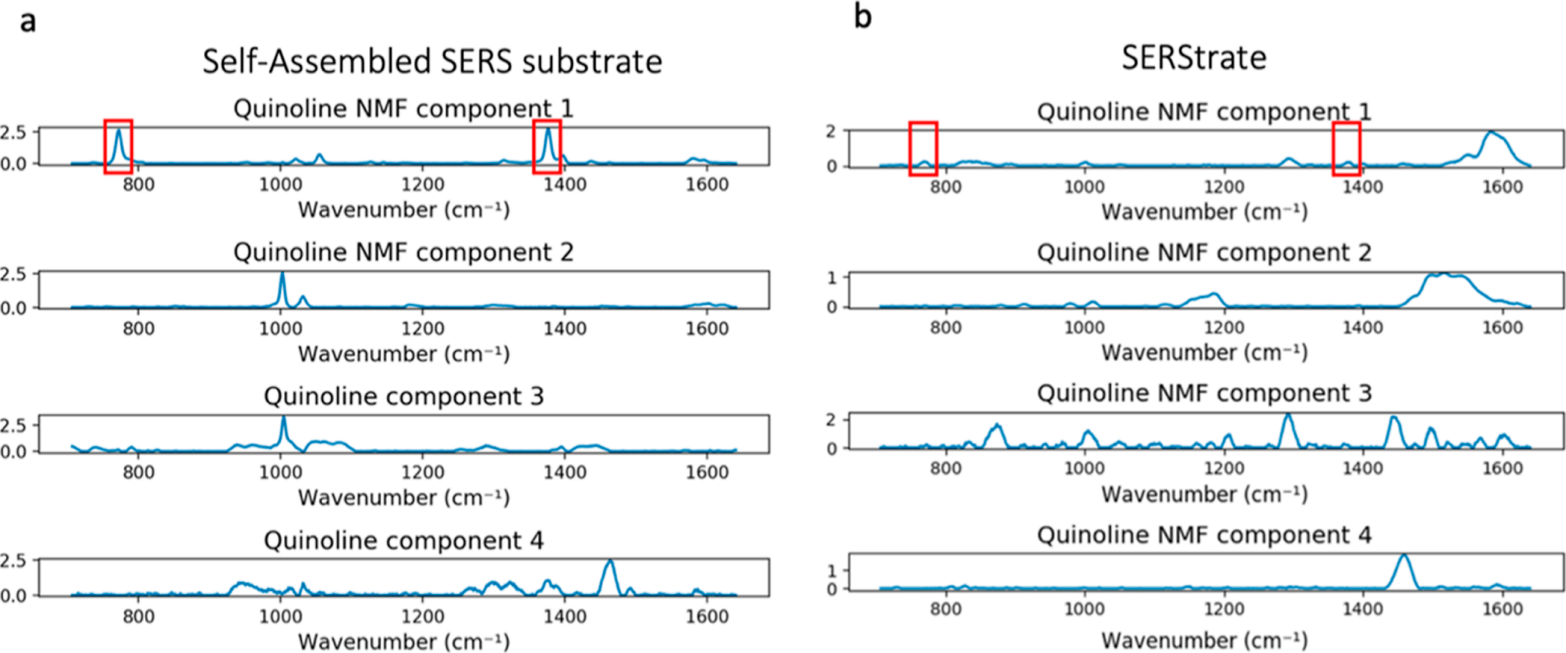
Four major NMF components separated from a complete Raman spectrum of wastewater seeded with quinoline and collected using the (a) self-assembled SERS substrate and (b) SERStrate.

Despite the reasonable quantification outcomes using a single characteristic Raman peak to detect quinoline concentrations in wastewater on the self-assembled substrate, the inclusion of multiple quinoline Raman scattered peaks in the analysis may further improve the performance for detecting target molecules as shown in the previous studies.^38,40^ The results shown in Figure 4 demonstrate the application of NMF filtering and the PLS regression model to separate the quinoline spectra among interference signals.

As seen in Figure 4a, NMF extraction of quinoline Raman signals yielded four major NMF components that could be separated from the Raman spectra of wastewater on the self-assembled SERS substrate. The first NMF component represents the quinoline characteristic Raman signal including two quinoline peaks located at 770 and 1376 cm^–1^, while the other three NMF components are attributed to the signals of the SERS substrate, background fluorescence, and minor contribution from molecules in the wastewater.

Figure 4b shows SERStrate Raman spectra separated by NMF. In the NMF component 1, two discernible quinoline Raman peaks were observed at 770 and 1376 cm^–1^. However, the peak intensity was significantly lower compared to that for the quinoline NMF component 1 in Figure 4a. Moreover, there were other interference peaks coexisting with quinoline signals in a single component, indicating that the NMF algorithm was limited due to the high background spectra noises detected in the wastewater and the weak intensification of quinoline signals. Although NMF filtered out most of the background spectral noise and enhanced the identification of quinoline signals, NMF could not resolve the issue of background interference, suggesting the importance of selective intensification of target molecules.

**Figure 5:**
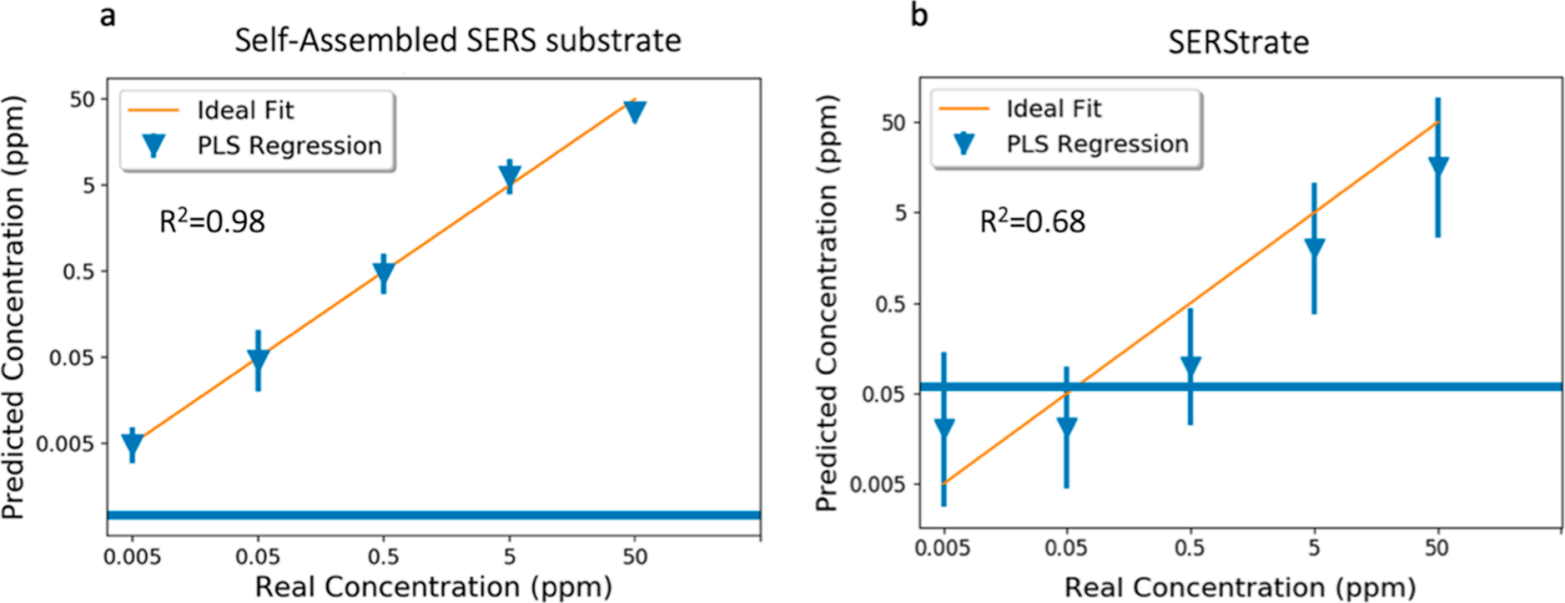
Quinoline concentration in spiked wastewater predicted by the PLS model. (a) Spectra collected using the self-assembled SERS substrate. (b) Spectra collected using SERStrate. The blue line indicates the PLS model-predicted quinoline concentration in unspiked wastewater, which represents the background noise from the interference molecules in the wastewater.

Quinoline concentrations predicted by the PLS model for quinoline-spiked wastewater collected using both the self-assembled SERS substrate and SERStrate are presented in Figure 5. 80% of spectral data were used for model construction, and the remaining 20% of the spectral data were used as a holdout set to test the accuracy of the predictive model. Figure 5a shows the relationship between spiked quinoline concentrations in treated wastewater and their predicted concentrations based on spectra collected with the self-assembled SERS substrate. The horizontal line shown in Figure 5 is the PLS model-predicted quinoline concentration of unspiked wastewater, which represents the background noise from the interference molecules in the wastewater.

A value of *R*^2^ = 0.98 shows good performance of the predictive model when analyzing the holdout set. In comparison with the linear regression curve built with the same data set using a single Raman peak (Figure 3c), there was a clear improvement in the predictive performance as indicated by an increase of *R*^2^ value from 0.94 to 0.98. Moreover, unlike the linear regression curve that overestimated the concentration of 5 ppb quinoline spiked in wastewater, the predictive model achieved an accurate prediction between 5 ppb and 50 ppm of quinoline spiked in wastewater.

Figure 5b displays the predictive result based on the spectra of quinoline-spiked wastewater collected using SERStrate. Despite the overall underestimation of quinoline concentrations, which might be affected by the coexisting of the noise peaks in the NMF component, a substantial improvement was observed in comparison with the linear regression curve built with the same data set (Figure 3d). Overall, the predictive model improved the quantification of quinoline at 500 ppb, 5 ppm, and 50 ppm, while the concentrations at 5 and 50 ppb were below the background noise. These results suggest that the predictive modeling using NMF and the PLS algorithm is useful for enhancing the sensitivity and accuracy of the detection method by analyte isolation followed by the multivariate analysis, even in a severe scenario with high background spectral noise.

### 3.4 Reusability of the SERS Substrate

**Figure 6:**
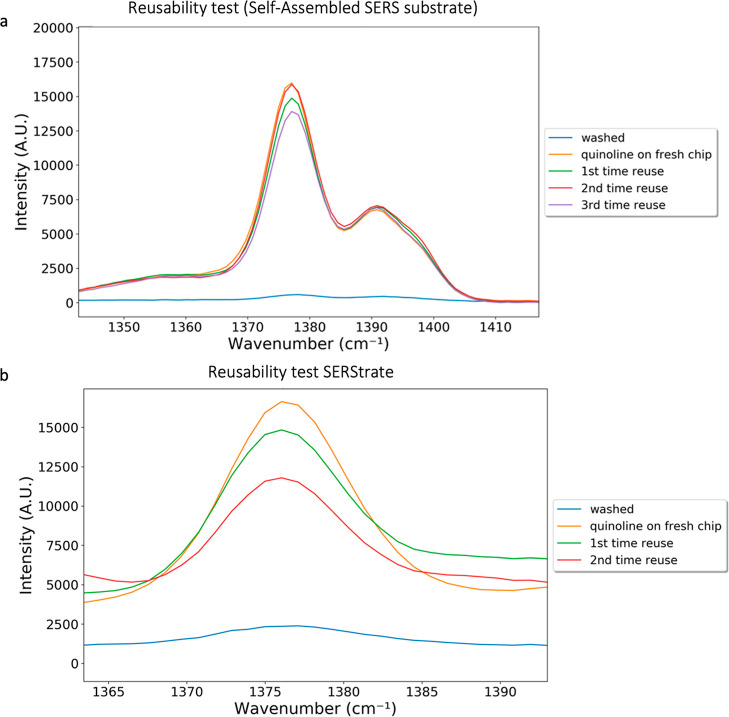
(a) SERS spectra of the washed self-assembled substrate and comparison of 50 ppm quinoline collected using freshly made and washed self-assembled SERS substrate at 1376 cm^–1^. (b) SERS spectra of acid-washed SERStrate and comparison of 50 ppm quinoline collected using fresh SERStrate and acid-washed SERStrate at 1376 cm^–1^.

On the self-assembled substrate, SERS signal enhancement relies on target molecules entering the hotspot region near the nanogap space through molecular diffusions in liquid; measurements are performed in water. The analyte can be easily removed from the substrate surface by DI water washing as shown in Figure 6a. No quinoline peak was found on the self-assembled SERS substrate after 20 min DI water washing. The reusability was demonstrated by the similar signal intensity of the quinoline peak when 50 ppm quinoline was loaded on virgin and washed self-assembled substrates (Figure 6a). The physical images of the self-assembled SERS substrate under the microscope before and after washes (Figure S4) also indicated that the self-assembled gold nanoparticles were stable during reuse.

SERStrate requires evaporation of the carrying fluid and the clasping of the gold nanofingers to achieve Raman signal enhancements. The substrate reusability results showed that DI water washing only was ineffective at removal of the signal of dried analytes from the SERStrate surface. However, washing by acid followed by rinsing with ethanol was able to recover the SERStrate surface (Figure 6b). The second round of sample preparation and drying revealed a similar Raman spectra intensity of the analyte (Figure 6b), suggesting recovery of gold nanofingers for recapturing the target molecules. However, the SERS capability degraded after the next round of the regeneration process, suggesting the deterioration of performance, which is likely due to the deformation of the gold nanofingers on the substrate surface.

## 4 Discussion

Real-time detection of antibiotics and other trace organic contaminants in wastewater is critical for human and environmental health protection. This research presents a proof-of-concept for the potential application of SERS as a label free, real-time sensor for monitoring of small-molecular-weight antibiotics in wastewater. Among various sensing techniques, Raman spectroscopy enables rapid, precise in situ molecular identification with the extremely low Raman activity of water molecules.^52^ SERS further expands the application scope of standard Raman spectroscopy and identifies molecules at the single-molecule scale.^53^ Because of such promises, many forms of the SERS substrate have been developed thanks to the rapid advancement of material sciences.^28,54^ However, different substrate designs affect the performance in different environments and for different targets.

Comparisons of results from the self-assembled substrate and SERStrate clearly show that SERStrate although can achieve great accuracy after capturing and immobilizing the target molecule within the nanogap space, the immobilization step limits the real-time potential of SERStrate. The evaporation of solvent to shrink the nanogap space of gold nanofingers is not only time consuming, but also the evaporation requirement limits the reusability of SERStrate due to the necessity of a more aggressive regeneration procedure to remove the dried chemicals trapped in the shrunken nanofingers. Aggressive cleaning caused the deformation of the gold nanofingers on the substrate surface. Therefore, SERStrate does not seem to have the potential to be incorporated into a real-time, reusable sensor design. On the contrary, the self-assembled substrate relies on molecular diffusion in the liquid around the gold nanoparticles on the surface with an evenly distributed nanogap space to achieve SERS. The self-assembled SERS substrate and SERStrate had a similar performance to effectively detect and quantify quinoline in pure water. The main advantage of the self-assembled substrate is the ease of removal of analytes from the surface by DI water washing for reuse in the next round of measurements. This allows a real-time sensor design by mounting a self-assembled SERS substrate in a testing chamber that is filled with a slow-flowing side stream of wastewater. The chamber is excited by a laser beam at pre-determined intervals, and the signal is captured by a spectrophotometer. A clean water wash stream to the chamber can be incorporated by switching the valves between the sample stream and the wash stream. The reusability study showed that there was no signal degradation after washing away the analyte and re-exposure. Gold nanoparticles were stably maintained on the surface. The proof-of-concept study presented here is to provoke thinking and future exploration of SERS application in real-time sensing of environmental contaminants.

Among various challenges facing SERS implementation as a label-free, real-time sensor for contaminants detection in a waste stream, interference from non-target molecules on the substrate surface is the Achilles’ heel of the technology. The results of this research found that the nanogap space between the gold nanoparticles on the SERS substrate played an important role in the selective intensification of the target molecules. SERS mainly depends on the electromagnetic effect, which is determined by the distance between metallic nanoparticles on the substrate, increasing with decreasing gap size. Single-molecule SERS intensity can be observed when nanogaps are on the order of 0.5–0.9 nm. In addition to the SERS enhancement factors, detection selectivity and reproducibility are also critical to ensure the performance of target detection in the wastewater environment. Self-assembled SERS substrates have a carefully designed gold-nanoparticle gap spacing of 0.9 nm with a high uniformity, which forms uniformly distributed electromagnetic hotspots over large areas, thus having both uniform and large enhancement factors across SERS substrates to reproducibly achieve low detection limits. Moreover, the self-assembled SERS substrate relies on molecular diffusion in the liquid around the nano-spacings on the surface, which excludes the interference from large molecules (size exclusion) that are commonly found in wastewater samples, and selectively enhances signals from small molecules within the designed nanogap spaces. Previous studies^38,40^ have shown that the self-assembled substrate can enhance Raman spectra of thiophenol (110.19 g/mol) and pyocyanin (210.236 g/mol) in addition to quinoline (129.16 g/mol) demonstrated in the current study. These chemicals have a small molecular size and a large Raman cross section. They can diffuse into the nanogap space to achieve SERS. Raman signal enhancement was not observed for larger molecular weight chemicals including humic acid, microcystin-LR, and erythromycin at concentrations greater than environmentally relevant concentrations. In addition to molecular sizes, the Raman cross section also plays an important role in SERS. Experiments with amino acids including glycine (75.07 g/mol) and l-arginine (174.2 g/mol) showed minimal interference at concentrations below 5 ppm, which is the approximate concentration level of amino acids in wastewater.^55^ These molecules are small enough to fit in the hotspots, but they have a low Raman cross section. This explains our observation of selective intensification of quinoline in wastewater without the interference of other wastewater molecules.

On the other hand, the commercial SERStrate platform requires drying to trap molecules within the nanofingers and to form the electromagnetic hotspots. Despite the promises of high enhancement factors and detection reproducibility, there is no selectivity for the molecular size of the target in the wastewater environment. Both large and small molecules can be trapped during the drying process, resulting in high interference by wastewater molecules. Therefore, the ideal SERS substrate for real-time environmental sensing requires (1) uniformed sub-nanometer nanogap dimensions over a large area; (2) selective SERS enhancement based on molecular size or other structure; and (3) SERS measurements in liquid environments to avoid side-effects of drying and to improve reusability of the substrate.

The coffee ring effect has been used to increase the sensitivity of SERS.^56^ However, the results using SERStrate suggest that the coffee ring formation following the evaporation of solvent causes uneven distribution of the Raman spectral intensity collected from randomly selected fields. The coffee ring formation depends on the time scale competition between liquid evaporation and the movement of suspended particles, and hence the particle size plays an important role. The coffee ring forms successfully only when the movement of the particles is faster than liquid evaporation. When the size of suspended particles is below 20 nm, the nanoparticles distribute uniformly onto the surface instead of forming a coffee ring structure after liquid evaporation.^57^ Therefore, the coffee ring was formed when the wastewater that contains many suspended particles > 20 nm was evaporated on the substrate but not in DI water which contains very low numbers of particles > 20 nm. The coffee ring decreases the reproducibility and accuracy of Raman measurements due to the non-uniform distribution of the target molecules. The unevenness of Raman spectral intensity also creates additional noises in data analysis, which further challenges the separation of Raman spectra of the target from the noise. Therefore, the commercial SERStrate platform that relies on the evaporation of solvent to create nanogaps has an unavoidable issue in signal uniformity. On the other hand, samples on the self-assembled SERS substrate do not form the coffee ring because the evaporation step is not required for Raman measurements. The Raman signal intensification is based on the molecular diffusion near the hotspots of the substrate, a key advantage for real-time measurement of target molecules.

Artificial intelligence (AI) was proposed as a potential solution to identify the target Raman signals among noises from interference molecules.^58^ Our previous work showed the successful separation of target Raman signals from background noises using a predictive model constructed with a machine learning algorithm for noise filtering.^38^ Here, we showed that NMF and PLS regression are powerful tools for analyzing the full Raman shift regime of the quinoline NMF component to keep the complete spectral information. We demonstrated the improvements in quinoline signal identification among high background noises in Raman spectra collected on SERStrate for quinoline-spiked wastewater. However, the model is still limited by the signal-to-noise ratio. More importantly, the interference molecules in the complex matrix, such as wastewater, not only generate overlapping Raman spectra with the analyte but also compete for the binding site for hotspots. On the other hand, synergy between the mechanism of the selective intensification of target molecules in the self-assembled SERS substrate and AI successfully identified the quinoline signal among complex matrices in the lowest spiked quinoline concentration. Therefore, the results of the comparative study indicate that the development of a real-time label-free sensor for residual antibiotic detection using SERS should start from the tunable design of the SERS substrate. The potential application of SERS in environmental sensing is exciting, but significant research is needed to transform the concept into a field-applicable technology.

## Supporting Information Available

Water quality parameters in the secondary effluent used for the seeding study; assignment of SERS bands of quinoline; comparison of SERS detection of quinoline in DI water with the quantification results by UPLC–MS/MS; PCA plot of SERS spectra collected in different scenarios; SERS spectra of chemicals with various sizes and Raman cross sections collected using the self-assembled SERS substrate; and microscopy images of fresh and washed self-assembled SERS substrate (PDF)
