# Supplementary material for: Sensing Antibiotics in Wastewater Using Surface-Enhanced Raman Scattering
Source: Environ Sci Technol. 2023 Mar 19;57(12):4880–91. doi: 10.1021/acs.est.3c00027 (PMC10061928; doi:10.1021/acs.est.3c00027)

Sensing Antibiotics in Wastewater using Surface Enhanced Raman Scattering

Yen-Hsiang Huang[1], Hong Wei[2], Peter Santiago[2], William John Thrift[2], Regina Ragan[2], Sunny Jiang[1, *]

[1]Department of Civil and Environmental Engineering, University of California, Irvine, Irvine, California 92697, United States

[2] Department of Materials Science and Engineering, University of California, Irvine, Irvine, California 92697, United States

Supplementary Information

Summary:

Number of pages: 4

Number of tables: 2

Number of figures: 4

Table S1. Water quality parameters in the secondary effluent used for seeding study

Parameter	Concentration	Description	Reference
Aluminum (mg/L)	19	Secondary effluent water quality based on 2008 and 2009 annual average data.	Project No. SP-173, Effluent Reuse Study GWRS Final Expansion FINAL Implementation Plan Volume 1 of 3
NH_3-N (mg/L N)	22.6		
Iron (mg/L)	350		
Manganese (mg/L)	45		
TDS (mg/L)	935		
TOC (mg/L)	14		
Turbidity (NTU)	3.2		
TBOD (mg/L)	11.1	Annual average activated sludge effluent biochemical oxygen demand for fiscal year 2004 -2005	FACILITIES OPERATION AND MAINTENANCE
CBOD (mg/L)	6.3		

Table S2. The assignment of SERS bands of quinoline

Experiment (cm^{-1})	Literature (cm^{-1})	Vibrational assignment
770	760	ring deformation
1019	1014	ring breathing
1030	1034	ring breathing
1057	-	CH bending
1133	-	CH bending
1264	-	CNC bending
1314	-	CH bending
1376	1372	CCC stretching
1391	1392	CCC stretching
1440	1433	CH rocking
1463	-	CH rocking
1579	1571	CCC stretching

Figure S1. Comparison of SERS detection of quinoline in DI water with the quantification results by UPLC-MS/MS.

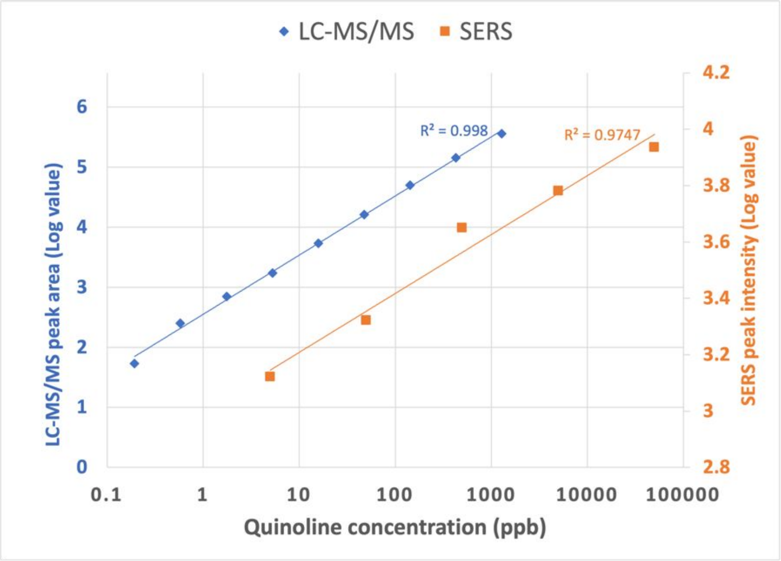

Figure S2. PCA plot of SERS spectra of DI water, treated wastewater, 50 ppm quinoline spiked in DI water or in treated wastewater. (a) Data collected on Self-Assembled SERS substrate; (b) Data collected on SEStrate.

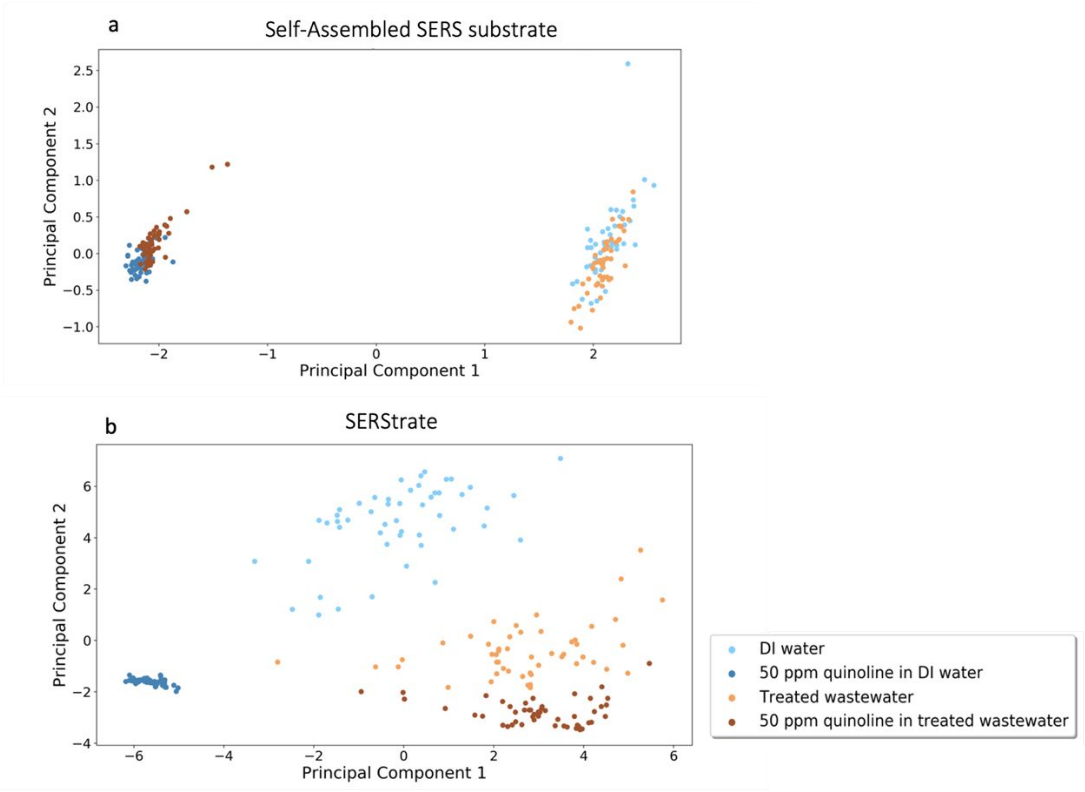

Figure S3. SERS spectra collected using Self-assembly SERS substrate for erythromycin, humic acid, Microcystin-LR, glycine, L-arginine and quinoline spiked DI water in the concentration of 5 ppm**.**

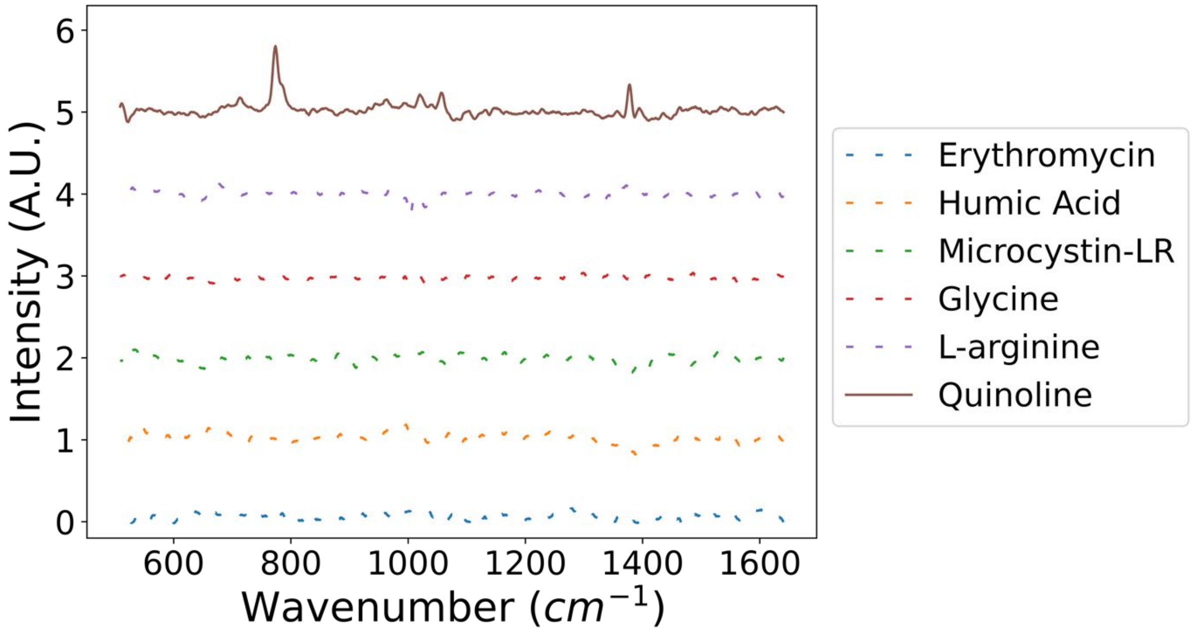

Figure S4. Microscopy image of (a) fresh Self-Assembly SERS substrate and (b) washed Self-Assembly SERS substrate.

a b

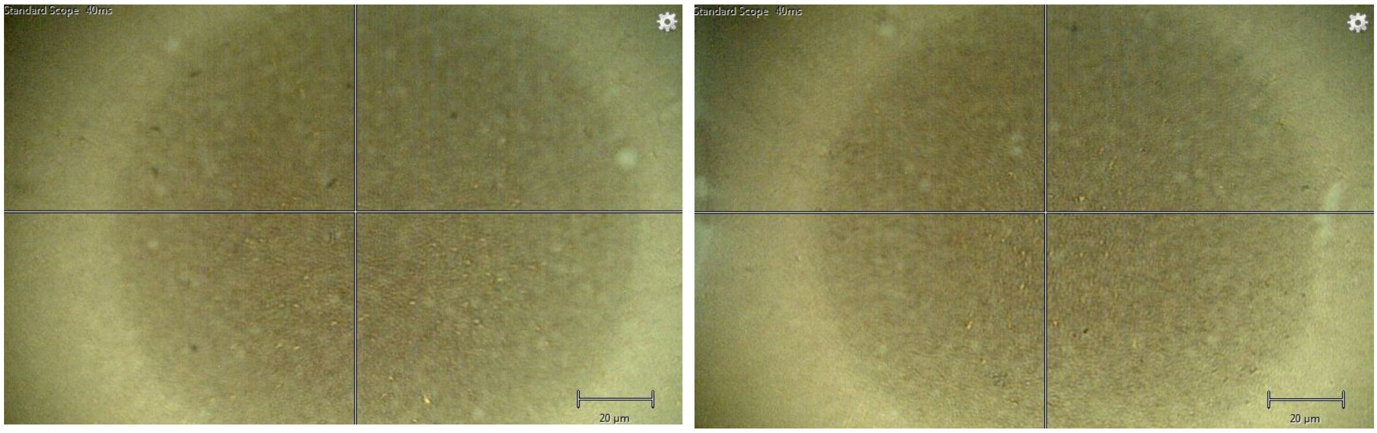

Supplement: Supplementary file 1 — es3c00027_si_001.pdf [file es3c00027_si_001.pdf]
